# TSPTFBS 2.0: trans-species prediction of transcription factor binding sites and identification of their core motifs in plants

**DOI:** 10.3389/fpls.2023.1175837

**Published:** 2023-05-09

**Authors:** Huiling Cheng, Lifen Liu, Yuying Zhou, Kaixuan Deng, Yuanxin Ge, Xuehai Hu

**Affiliations:** College of Informatics, Hubei Key Laboratory of Agricultural Bioinformatics, Huazhong Agricultural University, Wuhan, Hubei, China

**Keywords:** transcription factor binding sites, DenseNet, core motif, biological interpretability, trans-species prediction

## Abstract

**Introduction:**

An emerging approach using promoter tiling deletion via genome editing is beginning to become popular in plants. Identifying the precise positions of core motifs within plant gene promoter is of great demand but they are still largely unknown. We previously developed TSPTFBS of 265 *Arabidopsis* transcription factor binding sites (TFBSs) prediction models, which now cannot meet the above demand of identifying the core motif.

**Methods:**

Here, we additionally introduced 104 maize and 20 rice TFBS datasets and utilized DenseNet for model construction on a large-scale dataset of a total of 389 plant TFs. More importantly, we combined three biological interpretability methods including DeepLIFT, *in-silico* tiling deletion, and *in-silico* mutagenesis to identify the potential core motifs of any given genomic region.

**Results:**

For the results, DenseNet not only has achieved greater predictability than baseline methods such as LS-GKM and MEME for above 389 TFs from Arabidopsis, maize and rice, but also has greater performance on trans-species prediction of a total of 15 TFs from other six plant species. A motif analysis based on TF-MoDISco and global importance analysis (GIA) further provide the biological implication of the core motif identified by three interpretability methods. Finally, we developed a pipeline of TSPTFBS 2.0, which integrates 389 DenseNet-based models of TF binding and the above three interpretability methods.

**Discussion:**

TSPTFBS 2.0 was implemented as a user-friendly web-server (http://www.hzau-hulab.com/TSPTFBS/), which can support important references for editing targets of any given plant promoters and it has great potentials to provide reliable editing target of genetic screen experiments in plants.

## Introduction

Transcription factors (TFs) can activate or suppress transcription of genes by binding to the specific DNA sequences that are known as transcription factor binding sites (TFBSs), thereby playing an important role in gene expression. With the popularization of genome editing technology such as CRISPR-Cas9, it is now possible to genetically manipulate a TFBS to study how a TFBS regulates gene expression and then indirectly affects quantitative traits in plants (Wallace et al., 2018). Recently, a breakthrough reported that deleting the core motif (a 5-10 bp conserved DNA fragment that largely contributes the binding event) within a TFBS of An-1 through promoter tiling deletion of the rice *IPA1* gene can improve rice yield (Song et al., 2022). Thus, the identification of TFBSs, especially their core motifs, is of great demand in plant community and has become a fundamental step for plant breeding 4.0 (Wallace et al., 2018).

With the rapid development of next-generation sequencing (NGS) techniques, a number of experimental methods, both *in vivo* and *in vitro*, have been developed for TFBS discovery. The *in vitro* methods mainly contain protein binding-microarrays (PBM) (Berger and Bulyk, 2009), systematic evolution of ligands by exponential enrichment followed by sequencing (SELEX-seq) (Jolma et al., 2010), and DNA affinity purification followed by sequencing (DAP-seq) (O’Malley et al., 2016). The *in vivo* methods primarily include chromatin immunoprecipitation sequencing (ChIP-seq) (Johnson et al., 2007), chromatin endogenous cleavage sequencing (ChEC-seq) (Zentner et al., 2015). In the last decade, these high-throughput technologies, particularly ChIP-seq, have produced a large amount of TF-DNA binding data from some big projects, such as Encyclopedia of DNA Elements (ENCODE) (Moore et al., 2020) and Roadmap Epigenomics (Kundaje et al., 2015). They have enabled new insights into gene expression regulation in human and mouse. Although these experimental methods are successful, the application of them is limited by the long experiment time and high cost. Thus, developing computational methods to predict TFBSs and their core motifs has become an urgent issue.

Based on existing abundant experimental data, a series of data-driven computational methods have been proposed for predicting TFBSs varying from simple pattern matching methods to more complex models. As TFBSs are degenerate sequence motifs (Stormo, 2000), early researchers generally adopted position weight matrices (PWMs) to represent TFBSs and established two main TF databases: JASPAR (Fornes et al., 2020) and TRANSFAC (Matys et al., 2006). Based on databases, pattern matching methods attempted to predict TFBSs by just scanning candidate sequences of interests, with a model derived from experimentally determined binding sites for TFs (Wasserman and Sandelin, 2004; Grant et al., 2011). However, these scanning-based methods have two main disadvantages: (a) they only consider the DNA sequence features, but do not consider the information of respond variables like TF binding and gene expression. This leads to a high false positive rate because many matched DNA fragments are chromatin inaccessible without regulation function; (b) they cannot learn position dependencies between individual motifs.

In addition to these PWMs-based methods, many machine learning-based methods also have been developed to predict TFBSs. For a famous example, LS-GKM (Lee, 2016) leveraged support vector machine (SVM) to identify putative TBFSs of TFs and discover novel core motifs. For another example, Tsai et al. (2015) employed random forest to determine the sequence features, histone modification features and DNA structure features as significant features in TFBS prediction. Recently, deep learning methods have shown impressive performance in many fields, such as natural language processing and computer vision, inspiring researchers to apply deep learning-based approaches to predict putative TFBSs and motifs (He et al., 2021). For example, DeepBind (Alipanahi et al., 2015), one of the earliest and well-validated methods, leveraged convolutional neural networks (CNNs) to predict the sequence specificity of TF-DNA binding data. DeepSEA (Zhou and Troyanskaya, 2015), another deep learning-based algorithm, also employed CNNs and multi-task learning to identify TF-DNA binding motifs and functional effects of noncoding variants from large-scale chromatin profiling data. DanQ (Quang and Xie, 2016), an improved model of DeepSEA, combined CNN with bi-directional long short-term memory (Bi-LSTM) to predict motifs and quantify the functional SNPs from sequences. These three deep learning methods achieve outstanding performance and are now considered the state-of-the-art methods.

In plant community, relevant studies have lagged behind (Lai et al., 2019). For the data resource, the first large scale TFBS dataset of 529 *Arabidopsis* TFs was produced by DAP-seq in 2016 (O’Malley et al., 2016). Another remarkable progress is the 104 maize TFBS datasets produced by ChIP-seq in 2020 (Tu et al., 2020). Although reported TFBS datasets from other plant species are scattered, an important advance of ChIP-Hub offers a new centralized resource of TFBS datasets covering more than 40 plant species (Fu et al., 2022). These works all provide rice data resource for data-driven studies to meet the demands of plant community. However, existing TFBS datasets are still the tip of the iceberg when compared with the whole TFs across all plant species. Therefore, how to make use of existing TFBS data from model plant species to solve TFBS prediction tasks of other plant species is the trans-species prediction bottleneck of the current plant research.

For the modeling advances, our group previously developed a docker image called TSPTFBS, which contains 265 *Arabidopsis* TFBS prediction models based on DeepCNN (Liu et al., 2021). It provided a new insight for the computational methods of TFBS prediction in plants and demonstrated that the established models are feasible for trans-species TFBS prediction in other plants. A recent progress of PlantBind developed an attention-based multi-label deep learning framework to simultaneously predict the potential binding sites of 315 *Arabidopsis* TFs (Yan et al., 2022), and another tool of ‘TDThub’ (Grau and Franco-Zorrilla, 2022), who was based on FIMO (Grant et al., 2011), is a webserver for quick and intuitive studies of plant gene promoters. However, deep learning-based prediction model can only predict long DNA fragments with several hundred base pairs (bp) and cannot identify the precise genomic location of its core motifs (5-20 bp) with base resolution. In short, on one hand, scanning-based computational methods like FIMO (Grant et al., 2011) has an obvious defect of high false positive rate; on the other hand, deep learning-based computational methods like TSPTFBS (Liu et al., 2021) have a shortcoming of low resolution of motif identification. This is the identification bottleneck of functional motifs of the current plant research.

Aim at these two bottlenecks, our current work will make the following attempts. (i) We additionally introduced 104 maize and 20 rice TFBS datasets and utilized DenseNet for model construction on a large-scale dataset of a total of 389 plant TFs. We hope transfer predictability will be improved based on more powerful models trained on more TFBS datasets. (ii) Based on the trained models, we will combine three interpretability methods including DeepLIFT (Shrikumar et al., 2017), *in-silico* tiling deletion (de Almeida et al., 2022), and *in-silico* mutagenesis (Alipanahi et al., 2015) to identify the potential core motifs which importantly contribute TF binding event. Our work will have important applications not only on the molecular mechanism analysis of plant gene expression regulation, but also on the practice for plant breeding 4.0─breeding by genome editing (Wallace et al., 2018; Gao, 2021).

## Material and methods

### Data sources and preprocessing

Here, we first downloaded large-scale datasets including ChIP-seq datasets for 104 Zea mays TFs (Tu et al., 2020) (https://www.ncbi.nlm.nih.gov/geo/query/acc.cgi?acc=GSE137972) and 20 Oryza sativa TFs (Fu et al., 2022) (https://biobigdata.nju.edu.cn/ChIPHub/), DAP-seq datasets for 265 *Arabidopsis* TFs (O’Malley et al., 2016) (http://neomorph.salk.edu/PlantCistromeDB).

For the positive samples of a given TF, we extended the peak region to both the left and the right by 250 bp from the summit position or the middle position. For the negative samples of each TF, we randomly selected the sequences with the same sequence length and numbers as the positive sample after excluding the positive sample information and histone modification information in the intergenic regions of *Zea mays*, *Arabidopsis* and *Oryza sativa* genomes respectively. The distribution of positive sample number of 389 TFBS datasets is shown in **Figure S1**. We provided all the positive samples of each of 104 *Zea mays* TFs, 265 *Arabidopsis* TFs and 20 *Oryza sativa* TFs as bed files format on GitHub at: https://github.com/liulifenyf/TSPTFBS-2.0/tree/main/Data.

For each TF from three species, we pooled positive samples and negative samples together, and used uniform random sampling to divide the whole datasets into two parts: 80% for training and 20% for independent testing.

### The overall architecture of DenseNet

**Figure 1:**
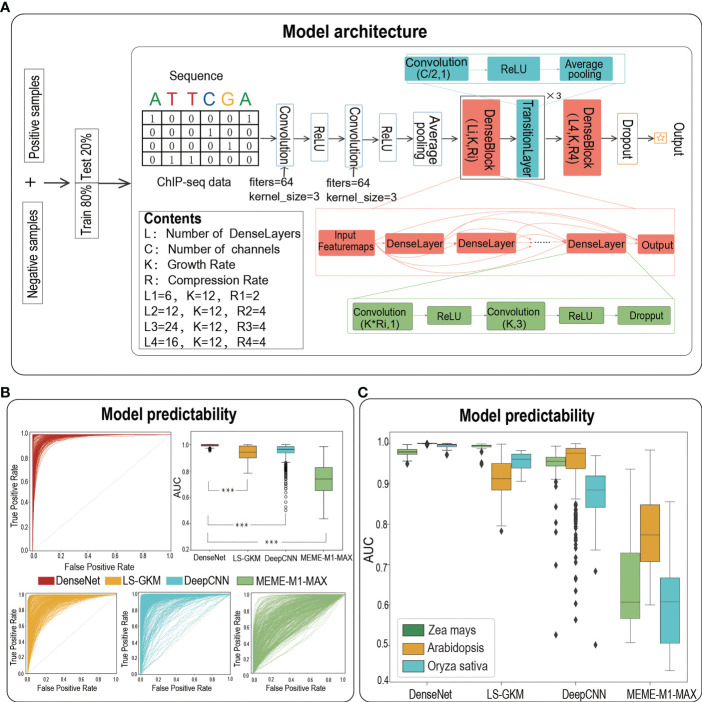
The architecture of deep learning model of DenseNet and the prediction performance. **(A)**The model architecture of DenseNet. **(B)** The ROCs and AUC distributions of DenseNet, LS-GKM, DeepCNN and MEME-M1-MAX on TFBS predictions for 389 TFs from three species. **(C)** The detailed AUC distributions of DenseNet, LS-GKM, DeepCNN and MEME-M1-MAX on three model plant species. *** represents a significant difference.

We then adopted a powerful architecture of DenseNet to build TFBS prediction models for each dataset. As the best paper award in CVPR-2017, DenseNet draws on the idea of ResNet to closely connect each layer with all the previous layers (Huang et al., 2016). It benefits from feature reuse to enhance feature propagation, thus alleviating the vanishing-gradient problem with depth deepening. The architecture of the DenseNet model employed in our study is composed of two convolution layers with the same number of convolution filters (filters = 64, kernel size = 3), an average pooling layer (stride = 2), four DenseBlocks and three TransitionLayers (**Figure 1A**). Each DenseBlock has L_i_ DenseLayers (L_i_ = 6, 12, 24, 16), which is composed of two convolution layers and all of these DenseLayers connect (with matching feature-map sizes) directly with each other. The TransitionLayer is mainly used to connect two DenseBlocks, including a convolution layer (kernel size = 1) and an average pooling layer (stride = 2). Finally, we flatten the output of the last DenseBlock and connect it to a fully connected layer to generate an output value. The maximum number of epochs to train was set to 80 and the learning rate was set to 0.001. We employed TensorFlow 2.0 to train models, and one can find the code at https://github.com/liulifenyf/TSPTFBS-2.0.

### Comparison of DenseNet models with other baseline methods

We compared the presented DenseNet models with other existing baseline methods, namely LS-GKM, DeepCNN, MEME-M1-MAX. For each method, we optimized the parameters and report the best results to perform a fair comparison. For LS-GKM, we first downloaded lsgkm from GitHub - Dongwon-Lee/lsgkm and then employed the function of ‘gkmtrain’ and the function of ‘gkmpredict’ with the default parameters. For DeepCNN, we designed the model including a convolution layer, a rectified linear unit (ReLU) layer, a max pooling layer and three fully-connected layers, and used sigmoid function for the output layer (Liu et al., 2021). The maximum number of epochs to train was set to 100 and the learning rate was set to 0.001. For MEME-M1-MAX, we used the training set of positive samples as the input of MEME-ChIP to extract the first five significant PWMs (with –meme-nmotifs 5) named M1, M2, M3, M4, and M5, and then employed six ways to score the test set of positive samples: M1-SUM (scan M1 and take sum of position scores), M1-MAX (scan M1 and take max of position scores), SUM-SUM (scan each M1.M5, sum over positions, then sum of five scores), MAX-SUM (scan M1.M5, max over positions, then sum of five scores), SUM-MAX (scan M1.M5, sum over positions, then max of five scores), and MAX-MAX (scan M1.M5, max over positions, then max of five scores) (Alipanahi et al., 2015). The results showed that when using the M1-MAX, the AUC for the most TF is higher than the other five ways. We finally selected M1-MAX as the final model and named it ‘MEME-M1-MAX’.

### Trans-species predictions

For a given TF from other species, we computed the protein sequence similarity between this TF and all the TFs of TSPTFBS and TSPTFBS 2.0 respectively *via* Blastp, and then utilized the prediction model of the most homologous TF (via alignment score and E-value< 1E-10) to perform trans-species prediction.

### Evaluation metrics

In this paper, the performance of all models were evaluated by ROC-AUC on the testing set to allow for fair comparisons with other existing baseline prediction methods. The performance measure is defined as follows:

ROC-AUC: The metric indicates the area under the receiver operating characteristic curve. It contrasts False Positive Rate (FPR) against True Positive Rate (TPR). A ROC-AUC value around 0.5 suggests a random classifier. The calculation method of TPR and FPR is defined as follows:


(1) $$TPR=\frac{TP}{TP+FN}$$

(2) $$FPR=\frac{FP}{FP+TN}$$

For the evaluation indexes of trans-species prediction, we used PPV (positive predictive value), NPV (negative predictive value) and recall as follows:


(3) $$PPV=\frac{TP}{TP+FP}$$

(4) $$NPV=\frac{TN}{TN+FN}$$

(5) $$recall=\frac{TP}{TP+FN}$$

TP (true positive) represents correctly predicted ‘positive’, TN (true negative) represents correctly predicted ‘negative’, FP (false positive) represents ‘negative’ incorrectly predicted as ‘positive’ and FN (false negative) represents ‘positive’ incorrectly predicted as ‘negative’.

### DeepLIFT

We employed a powerful interpretability tool called ‘DeepLIFT’, a new approach to assign importance score to the inputs for a given output. Specifically, DeepLIFT first compared the prediction values of the given sequences and their reference sequences (The reference sequence refers to dinucleotide shuffle based on the input sequence) and then assigned a DeepLIFT contribution score to each base of a given sequence according to decompose the difference by a backpropagation-like algorithm (Grau and Franco-Zorrilla, 2022). One can find the DeepLIFT code at https://github.com/liulifenyf/TSPTFBS-2.0/blob/main/interpretability.py.

### *In-silico* tiling-deletion

We further employed *in-silico* tiling-deletion (de Almeida et al., 2022) which simulates the experimental process of gene editing with high coverage of the whole sequence to identify those regions that have great influences with binding intensity after their deletions. Specifically, it was performed by scrambling the nucleotides within 10 bp windows with 1 bp steps. We employed ‘N’ to replace the scrambled nucleotides and computed the predicted value of the whole sequence edited with ‘N’. One can find the In-silico tiling-deletion code at https://github.com/liulifenyf/TSPTFBS-2.0/blob/main/interpretability.py.

### *In-silico* mutagenesis

We used *in-silico* mutagenesis, in which every mutation to a sequence is tested, a powerful approach for dissecting the exact nucleotides driving a binding activity to compute the predicted binding intensity (the binding probability predicted with model) of all possible mutations to a sequence (Alipanahi et al., 2015; Kelley et al., 2016). We drew a heatmap that display the change in predicted binding intensity from mutation at each position to each alternative nucleotide. One can find the in-silico mutagenesis code at https://github.com/liulifenyf/TSPTFBS-2.0/blob/main/interpretability.py.

### TF-MoDISco

A number of motif discovery methods for neural network have been produced in recent years, such as DeepBind (Alipanahi et al., 2015), Basset (Kelley et al., 2016). These methods only analyze individual filters and do not explain the fact that neural network learn distributed representations where multiple neurons cooperate to describe a single pattern, leading found patterns redundant. A new motif analysis tool of TF-MoDISco can generate high-quality, non-redundant motifs which takes DeepLIFT contribution scores as the input and then recognizes segments with substantial contributions that higher than background distribution of scores as ‘seqlets’ (Shrikumar et al., 2018). One can then align the identified seqlets into known TF motifs in large-scale databases such as JARSPAR.

The parameters used in this study are:

target_seqlet_fdr=0.15;

sliding_window_size=15;

flank_size=5;

final_min_cluster_size=60.

And the code used to perform TF_MoDISco can be found at https://github.com/liulifenyf/TSPTFBS-2.0/blob/main/modisco_test.py.

### Global importance analysis

Unlike the previously proposed model interpretability approaches for deep learning, such as attribution methods, global importance analysis (GIA) quantifies the population-level effect size that identified potential core motifs have on model predictions (Koo et al., 2021). GIA provides a natural follow up to current interpretability methods to quantitatively test hypotheses of identified potential core motifs (and their interactions with other patterns). In our study, we employed the GIA to quantify the effect size of motif identified by TF-MoDISco. More specifically, for each core motif identified by TF-MoDISco, we embedded the DNA sequence fragment with the top affinity according to the annotated PWM, into the different positions of the 70^th^ bp, the 170^th^ bp, the 270^th^ bp, the 370^th^ bp and 470^th^ bp within 1000 negative samples and predicted their binding probability with the trained model. The original predicted value was subtracted from the average probability across the different positions to obtain the local importance. The resultant local importance was averaged across all 1000 negative samples to derive the final global importance of each motif. Furthermore, we embedded all possible single nucleotide mutants of the top affinity sequence of seqlet1 (GCACGTGC) in 1000 negative samples to calculate their global importance and drew a heatmap of their differences of global importance with a sequence logo that has heights scaled according to the L2-norm at each position.

### Distribution pattern of six enriched TF motifs of bHLH145

To demonstrate distribution pattern of six enriched TF motifs, we calculated the motif occurrence frequency of six TF motifs within bHLH145 *via* a R package of ‘motifmatchr’ with the following parameters: p.cutoff=1e-05 (Schep, 2021). More specifically, for each motif, we first counted the total instance number as the occurrence number of this motif and then mapped the position of all instances of the selected motif within the positive samples of bHLH145 *via* the function of ‘matchMotifs’ in the R package of ‘motifmatchr’. As a result, we gave the matrix of occurrence number of each positive in **Table S3**.

## Results

### DenseNet has achieved greater predictability than other baseline methods

We previously have verified that the prediction performance of TSPTFBS is superior to other methods on 265 *Arabidopsis* TFBS datasets. We now naturally ask whether TSPTFBS 2.0, which both integrated additional 124 TFBS datasets (104 *Zea mays* and 20 *Oryza sativa*, a total of 389 datasets) and employed a new model architecture of DenseNet (Huang et al., 2016) (**Figure 1A**), will perform better prediction. Expectedly, DenseNet substantially outperformed the previous approaches by achieving a median AUC of 0.9997 on the test set of a total of 389 datasets, while LS-GKM (Lee, 2016), DeepCNN (Liu et al., 2021) and MEME-M1-MAX obtained a median AUC of 0.943 (t.test, P-value=2.583E-78), 0.963 (t.test, P-value=5.016E-41) and 0.739 (t.test P-value=3.248E-203) respectively (**Figures 1B, C**; **Table S1**). More precisely, the median AUC value of DenseNet in *Zea mays*, *Arabidopsis* and *Oryza sativa* were 0.979, 0.9999 and 0.996 respectively. LS-GKM achieved a slightly better predictability in *Zea mays* (median AUC 0.991), but in *Arabidopsis* and *Oryza sativa* it was significantly inferior to DenseNet (median AUC 0.912 in Arabidopsis and 0.961 in Oryza sativa). As a summary, this demonstrates that DenseNet has achieved greater predictability than other baseline methods on a total of 389 plant TFBS datasets from three plant model species.

### TSPTFBS 2.0 integrating more datasets is superior to TSPTFBS on trans-species predictions

We next ask whether TSPTFBS 2.0 which integrated more datasets will perform better than TSPTFBS 1.0 on trans-species TFBS predictions. To this end, we conducted a comparative analysis of trans-species TFBS predictions between them under the idea of transfer learning (Pan and Qiang, 2010). Firstly, we collected ChIP-seq datasets of 15 TFs covering other six species from ChIP-Hub (Fu et al., 2022) (including each 1 for *Arabis alpina*, *Chlamydomonas reinhardtii*, *Hordeum vulgare*, *Physcomitrella patens*, 2 for *Eucalyptus grandis*, and 9 for *Solanum lycopersicum*) and then compared each TF prediction performance with TSPTFBS 1.0 and TSPTFBS 2.0 respectively.

**Figure 2:**
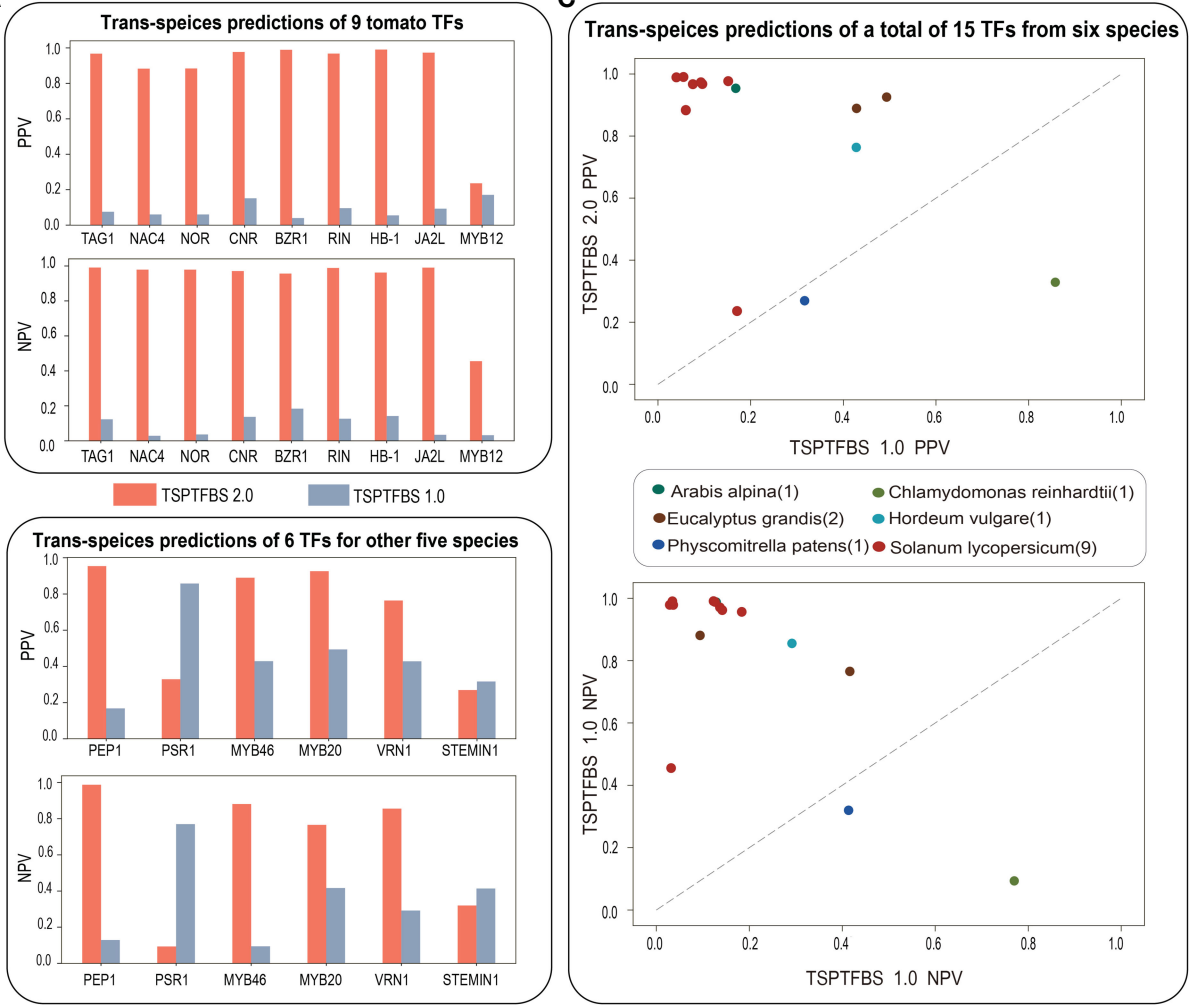
The trans-species prediction performance. **(A)** The trans-species prediction performance (via PPV and NPV) of nine tomato TFs. **(B)** The trans-species prediction performance (via PPV and NPV) of six TFs for other five species. **(C)** The comparative analysis on trans-species TFBS prediction between TSPTFBS 2.0 and TSPTFBS 1.0 on a total of fifteen TFs covering six species (via PPV and NPV).

For the result, 100% (9 out of 9) of tomato TFs in TSPTFBS 2.0 achieved greater positive predictive values (PPVs) and negative predictive values (NPVs) than TSPTFBS 1.0, and 88.89% (8 out of 9) of tomato TFs achieved satisfied PPVs and NPVs between 0.8 and 1.0 (**Figure 2A**). For another 6 TFs from other five species, 66.7% (4 out of 6) of TFs in TSPTFBS 2.0 achieved greater PPVs and NPVs than TSPTFBS 1.0, and 50% (3 out of 6) of TFs achieved satisfied PPVs and NPVs between 0.8 and 1.0 (**Figure 2B**). In summary, 86.67% (13 out of 15) TFs of TSPTFBS 2.0 yielded greater PPVs and NPVs than those of TSPTFBS 1.0 (**Figure 2C**, we listed PPVs and NPVs of 15 TFs with TSPTFBS 2.0 and TSPTFBS 1.0 in **Table S2**), implying that TSPTFBS 2.0 trained with more powerful model on more TFBS datasets is superior to TSPTFBS 1.0 on trans-species predictions.

### A useful strategy of combining three biological interpretability methods to identify potential core motifs within a TFBS

There has been a new trend that plant scientists have employed tiling-deletion-based CRISPR–Cas9 strategy to screen a gene promoter and to identify its core motifs, which significantly affect crop traits through regulating the expression of target gene, in plant breeding (Song et al., 2022). We next ask whether we can identify core motifs importantly contributing TF binding events, in turn regulating target gene expression. To this end, we next explore a new application, which is also a highlight of our current research: identifying potential core motifs that significantly affect TF binding by the following interpretability analysis.

**Figure 3:**
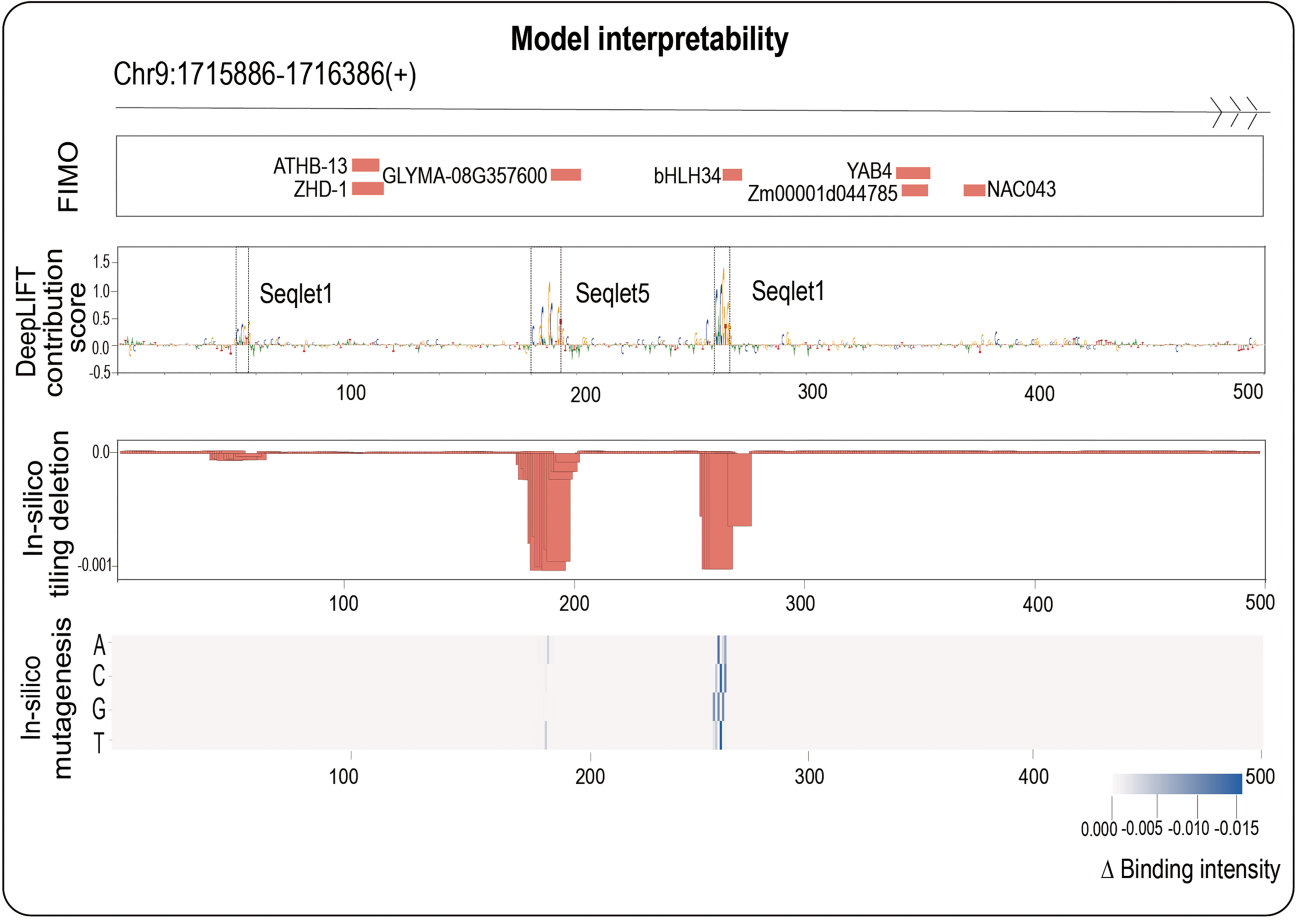
One positive in bHLH145 is used as an example to illustrate the ability to identify core motif of our work. The top panel shows the sequence region: Chr9:1715866-1716386; the second panel shows the FIMO results; the third panel shows the DeepLIFT base contribution scores; the fourth panel shows the result of *in-silico* tiling deletion which compute the difference of predicted value with a sliding window of 10 bp deletion across the whole sequence; at the bottom, a heatmap showing the *in-silico* mutagenesis result.

Here, we adopted three mainstream interpretability methods: backpropagation-based DeepLIFT (Shrikumar et al., 2017), perturbation-based *in-silico* tiling deletion (de Almeida et al., 2022) and *in-silico* mutagenesis (Alipanahi et al., 2015) from different perspectives (see Materials and methods section for more details). We next naturally ask whether combining three interpretability methods can identify potential core motifs within a TFBS accurately. We here focus on maize bHLH145 TF (Zm00001d031717), and randomly select an example sequence (Chr9: 1715886-1716386(+)) from its positive samples to demonstrate the results of three methods (**Figure 3**): we found that DeepLIFT has detected three regions (53-58 bp, 182-193 bp and 262-267 bp) with successive high contribution scores, however, we cannot judge whether those regions have great influences with binding intensity after their deletions. Further combining the *in-silico* tiling deletion result, we can clearly find that two of them have obvious editing significance, and the *in-silico* mutagenesis result further indicated the mutation directions with the strongest editing effects which relate to TF binding at every base (**Figure 3**). For comparison, we also performed FIMO which scanned the given DNA fragment to find motifs having good match with motifs in database like MEME (Bailey et al., 2009). For the result, we found that the regions detected by three interpretability methods are truly known motifs (such as bHLH34 in the center). In contrast, the other motifs detected by FIMO are ATHB-13, ZHD-1 and NAC043 (**Figure 3**), whose function need to be studied in the future. These findings imply that we can accurately identify the potential core motifs within a TFBS by combining three biological interpretability methods.

TF motif analysis with TF-MoDISco and global importance analysis

**Figure 4:**
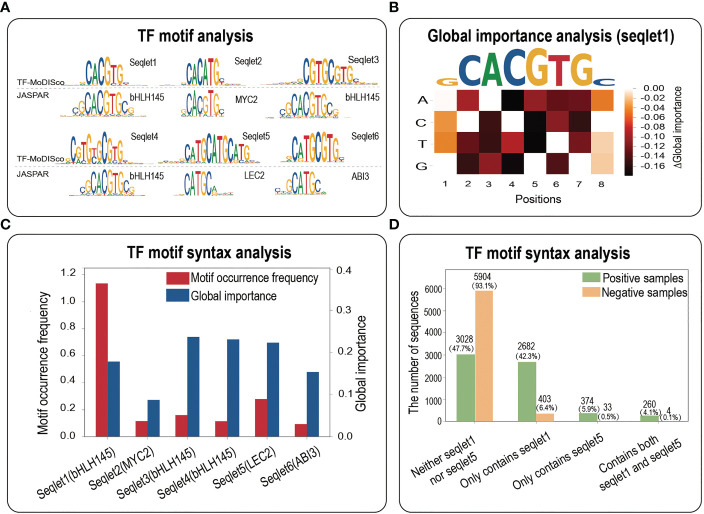
TF motif analysis. **(A)** The motif analysis based on all the positives of bHLH145. Top panel, a comparative result of motif identified by TF-MoDISco and known motif in JASPAR. **(B)** A heatmap of the difference in the global importance for 1,000 random negative samples embedded with single nucleotide mutations of the DNA sequence fragment corresponding to the top affinity (‘GCACGTGC’). **(C)** Motif occurrence frequencies and global importance values of 6 seqlets identified by TF-MoDISco in the bHLH145 positives. **(D)** Motif occurrence numbers of four cases (neither seqlet1 nor seqlet5, only seqlet1, only seqlet5 and both seqlet1 and seqlet5) in bHLH145 positive and negative samples.

To investigate the biological implications of potential core motifs identified by three interpretability methods, we employed a motif discovery algorithm named TF-MoDISco to identify high-quality and non-redundant TF motifs (Shrikumar et al., 2018). Based on all the positive samples of maize bHLH145 TF, we totally identified 9 seqlets, 6 out of which were perfectly matched to the JASPAR database (**Figure 4A**). Expectedly, seqlet1 was mapped into the bHLH145 motif in JASPAR (**Figure 4A**), implying that our model has learned the core bHLH145 motif. To further explore whether the identified core TF motifs are universally functional, we performed the global importance analysis (GIA) to quantify their effect sizes in the random context of negative samples (Koo et al., 2021). We found that the core motif obtained by GIA is highly consistent with it in JASPAR (**Figure 4B**), which indicates that the core motif identified by TF-MoDISco also has important effects in a random background, further demonstrating its sufficiency for TF binding.

More interestingly, seqlet5 was mapped into the LEC2 motif and has a high global importance (**Figures 4A, C**), which suggests that the TF binding might involve cooperative TF motifs. We then calculated motif occurrences of four cases (neither seqlet1 nor seqlet5, only seqlet1, only seqlet5 and both seqlet1 and seqlet5) in positive and negative samples respectively *via* a motif scanning (**Table S3**). Expectedly, seqlet1 dominates the contributions and appears in nearly a half of positive samples [(2682 + 260)/6344≈46.4%)], and seqlet5 appears in a small number of positives [(374 + 260)/6344≈9.99%)], implying its assistant function. Interestingly, their co-occurrence in positives (260/6344≈4.1%) is far greater than that of negative samples (4/6344≈0.063%) (**Figure 4D**), which further suggests that there is a functional interaction between the two seqlets in some positives and implies the necessity of their co-occurrence for TF binding. Overall, the TF motif analysis demonstrates that interpretability methods not only can identify core motifs within a TFBS sample, but also have potential contributions in revealing important TF motif syntax such as motif co-occurrence.

### A webserver as an application for discovering potential core motifs within any given plant promoters

Although our method can theoretically detect core motifs of any genomic region, we focus on plant promoter region in view of its high importance. Finally, we developed a pipeline of TSPTFBS 2.0, which integrates 389 DenseNet-based models of TF binding and the above three interpretability methods. This pipeline was implemented as a user-friendly web-server (http://www.hzau-hulab.com/TSPTFBS/) for discovering potential core motifs within any plant promoters. More precisely, for a given TF model of interest, we allow the user to upload the promoter sequence (upstream 500 bp of plant gene TSS of interest) in our website, and the web-server will return the following results: (i) the predicted binding intensity of the uploaded promoter sequence of the given TF; (ii) numeric results of three interpretability methods—base contribution score from DeepLIFT, the difference of predicted value between WT (Wild Type) and the sequence after a 10 bp deletion from *in-silico* tiling deletion and the difference of predicted value between WT and the sequence after a point mutation from *in-silico* mutagenesis; (iii) a logo graph for intuitively displaying the above interpretability results.

**Figure 5:**
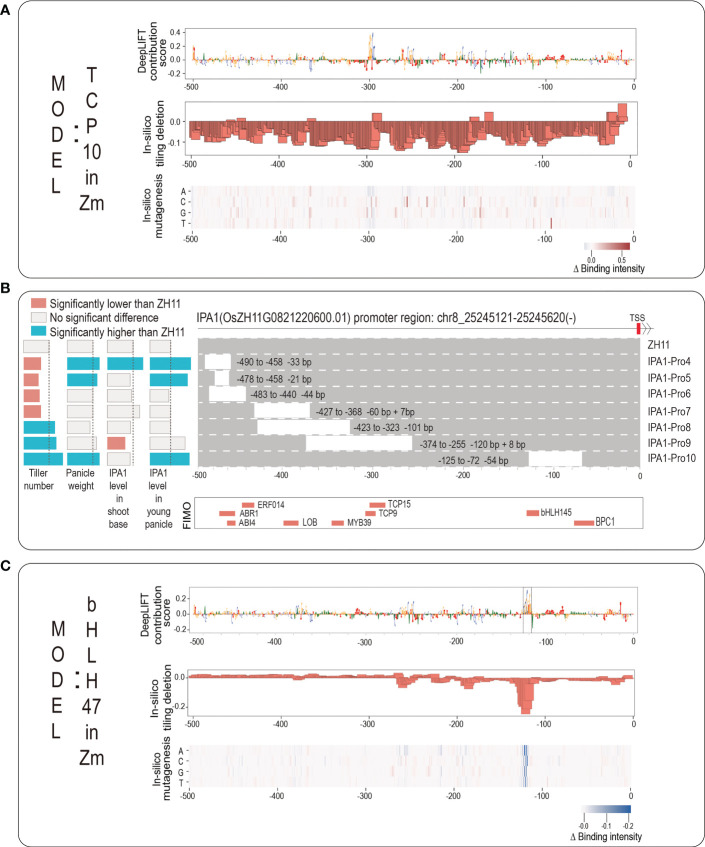
The application for discovering potential core motifs based on rice *IPA1* promoter. **(A)** The logo graph for intuitively displaying the interpretability results of TCP10 model in Zea mays. The top panel shows the DeepLIFT base contribution scores; the second panel shows the result of *in-silico* tiling deletion; at the bottom, a heatmap for showing the *in-silico* mutagenesis result. **(B)** The top panel shows the *IPA1* promoter region: Chr8:25245121-25245620; the second panel shows the experimental results from published work (Song et al., 2022) including: seven editing events relative to the ZH11 WT and their corresponding changes in IPA1 gene expression and two phenotypes of tiller number and panicle weight; the third panel shows the scanning results by FIMO; **(C)** The logo graph for intuitively displaying the interpretability results of bHLH47 model in Zea mays.

We next take an example of rice *IPA1* gene promoter for a detailed demonstration. *IPA1* is a key rice gene that is a master regulator of rice plant architecture. Its function was known to increase grains per panicle but reduce tillers; however, a recent breakthrough showed that a 54-base pair (covering a critical TFBS of An-1) *cis*-regulatory deletion can increase both grains per panicle and tiller number (Song et al., 2022). An-1 binds to the binding motif of ‘GCGCGTGT’ and it is a basic helix-loop-helix transcription factor (bHLH TF), which positively regulated awn length and negatively regulated grain number per panicle (Luo et al., 2013). To investigate whether our novel strategy can identify this An-1 motif without the prior knowledge of bHLH TF, we first employed FIMO to scan the promoter sequence of *IPA1* (from -500 to TSS) and showed the scanning results in **Figures 5B**; **S2B**, in which a total of nine motifs, including bHLH145, TCP9, TCP15, have been detected by FIMO. This implies that the promoter sequence of *IPA1* has the above sequence motifs. And then we searched our 389 TF models to find the corresponding TFs with the same family of each of the above nine TFs. For bHLH TF family, there are a total of 14 TF models (10 bHLH *Zm* TFs and 4 bHLH *At* TFs). We plotted the predicted binding intensity of the promoter sequence of IPA1 from each of the 14 bHLH TF models in **Figure S2A**, and we chose the model of bHLH 47 in *Zm* to perform interpretability analysis. For TCP TF family, there are two TCP TF models of TCP 10 in *Zm* and TCP 23 in *Zm*, and we chose the model of TCP 10 in *Zm* to perform interpretability analysis.

We input the promoter sequence of *IPA1* into the two TF models of TCP10 in *Zm* and bHLH47 in *Zm*, and drew the corresponding logo graphs of their interpretabilities in **Figures 5A, C**. As it can be seen from **Figure 5A**, although an obvious peak (-299 to -293, corresponding to TCP10) with successive high contribution scores appears in the position near -300, the results from *in-silico* tiling deletion and *in-silico* mutagenesis do not strongly support it. As the experimental result (Song et al., 2022), we found that the editing event of *IPA1*-Pro9 (this editing event has a deletion of the above region) only significantly decreased the *IPA1* level in shoot but not in young panicle, which leads a significant increasement of tiller number but no significant change of panicle weight. From **Figure 5C**, we found that all three interpretability methods all support the importance of a peak (-124 to -117, corresponding to bHLH47) near the position near -125, and we surprisingly found that it can highlight the ‘GCGCGTGT’ motif located from -128 to -117 (marked with dashed box). Actually, the editing event of *IPA1*-pro10 (it has a deletion of this peak) leads to increasements both in tiller number and in panicle weight. As for other scanning motifs by FIMO, we found no Zm TF models but only found At TF models. And we drew them in **Figure S2C** and found no obvious clues about the core motifs. In summary, the example of *IPA1* implies that our webserver is helpful for identifying core motifs within plant gene promoter and for supporting references of gene editing.

## Discussion

Identifying the precise positions of core motifs within gene promoter is of great demand in plants because they are the potential editing targets. These core motifs are often TFBSs affecting gene expressions. However, current plant research involving TFBS has two research bottlenecks: the trans-species prediction bottleneck and the identification bottleneck of functional motifs. In this paper, we developed a large-scale TFBS models from three model plant species of *Arabidopsis*, maize and rice and have proven that trans-species prediction on 15 TFs from other six plant species is feasible. We also developed three interpretability methods to identify base-resolution core motif. Finally, we took rice *IPA1* gene (Song et al., 2022) as an example to discuss how to employ three interpretability methods to contribute practical applications in plant breeding.

## Conclusions

In conclusion, TSPTFBS 2.0 used DenseNet to improve the predictability of TFBS prediction, and further verified that the trans-species TFBS prediction capability of TSPTFBS 2.0 was significantly improved, which is the first contribution of the current work. The main contribution is that we combined three interpretability methods to identify the potential core motif within a TFBS, which will be a powerful tool for assisting plant scientists on providing candidate targets of genome editing. To be convenient for applications, TSPTFBS 2.0 integrates 389 DenseNet-based models of TF binding and three interpretability methods, and it was implemented as a user-friendly web-server (http://www.hzau-hulab.com/TSPTFBS/), which has great potentials to provide reliable editing target of genetic screen experiments in plants.

## Data availability statement

The datasets presented in this study can be found in online repositories. The names of the repository/repositories and accession number(s) can be found in the article/**Supplementary Material**.

## Author contributions

HC and LL: Data curation, formal analysis, and writing original draft preparation. YZ, KD and YG: Formal analysis. XH: Conceptualization, funding acquisition, and review and editing. All authors contributed to the article and approved the submitted version.
