# Supplementary material for: TSPTFBS 2.0: trans-species prediction of transcription factor binding sites and identification of their core motifs in plants
Source: Front Plant Sci. 2023 May 9;14:1175837. doi: 10.3389/fpls.2023.1175837 (PMC10203575; doi:10.3389/fpls.2023.1175837)
Supplement: Supplementary file 1 [file DataSheet_1.docx]

**Supplementary Information**


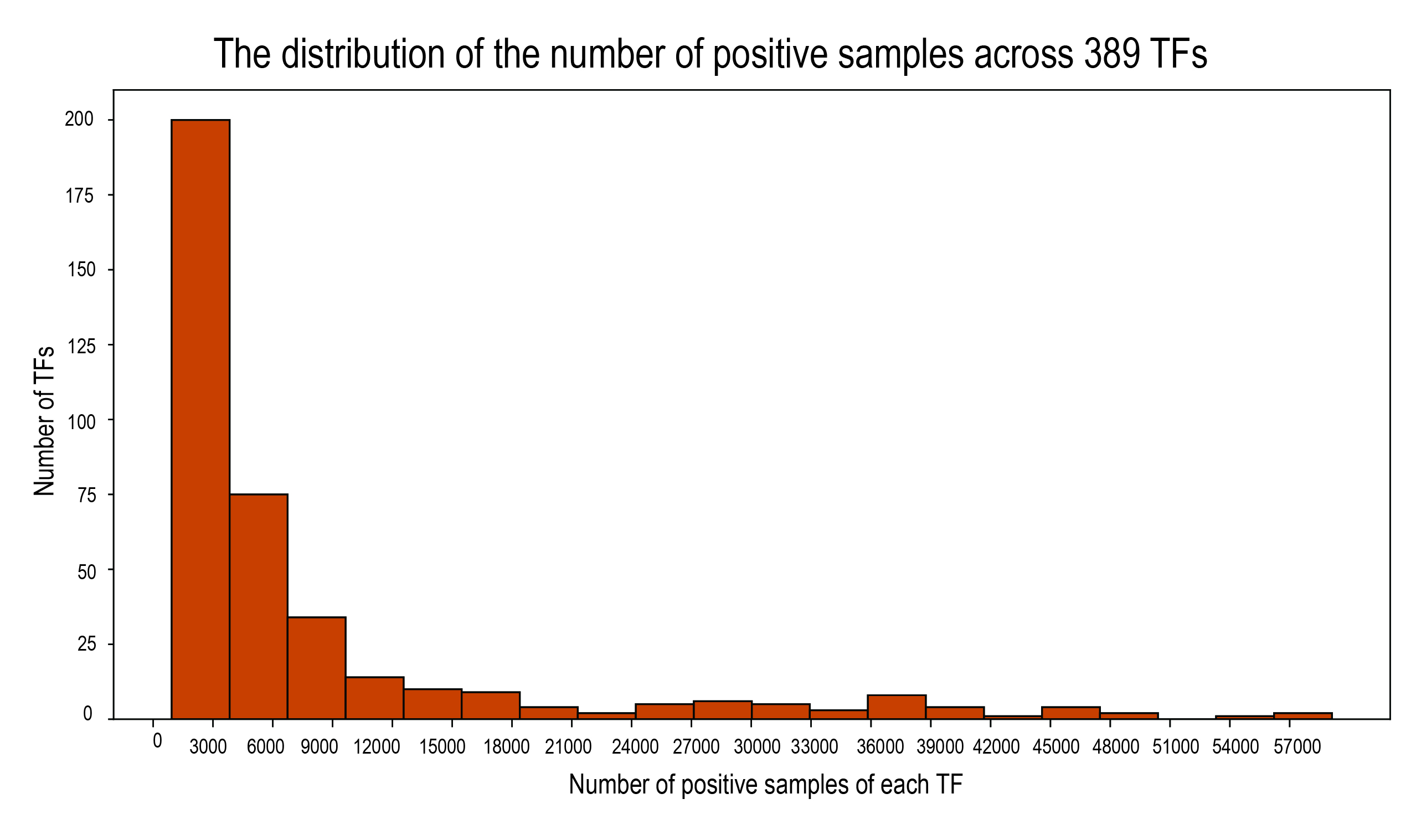
**Supplementary Figures:**

**Figure S1.** The distribution of positive sample number of 389 TFBS datasets including 104 *Zea mays* TFs, 265 *Arabidopsis* TFs and 20 *Oryza sativa* TFs.


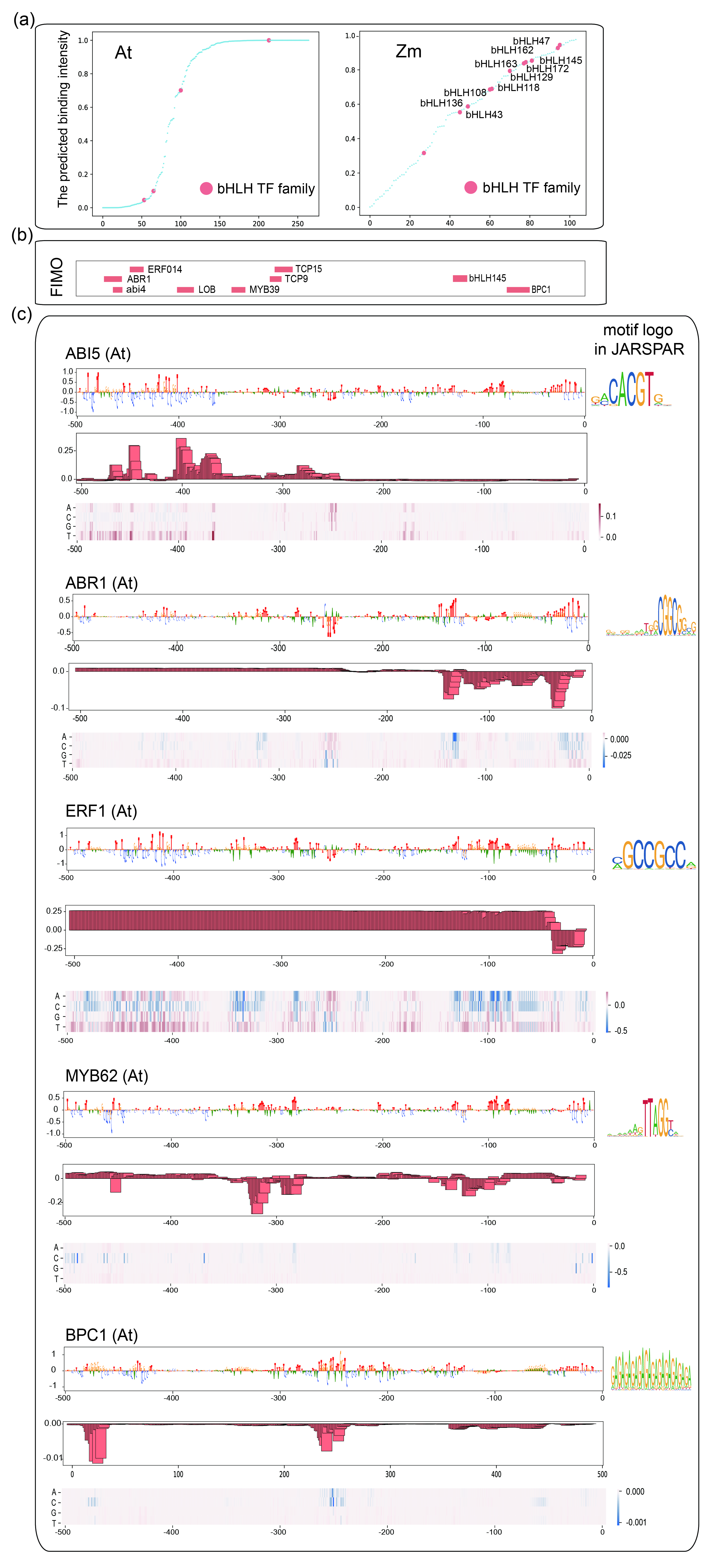
Figure S2. The scanning results by FIMO and the corresponding interpretability results with five At TF models of ABI5, ABR1, ERF1, MYB62 and BPC1.
